# Nanoparticle‐based applications for cervical cancer treatment in drug delivery, gene editing, and therapeutic cancer vaccines

**DOI:** 10.1002/wnan.1718

**Published:** 2021-05-04

**Authors:** Peijie Zhou, Wei Liu, Yong Cheng, Dong Qian

**Affiliations:** ^1^ Department of Radiation Oncology, The First Affiliated Hospital of USTC, Division of Life Sciences and Medicine University of Science and Technology of China Hefei Anhui China

**Keywords:** cervical cancer, CRISPR, drug delivery, nanoparticle, therapeutic vaccine

## Abstract

Cervical cancer is a leading cause of gynecological tumor related deaths worldwide. The applications of conventional approaches such as chemoradiotherapy and surgery are restricted due to their side effects and drug resistances. Although immune checkpoint inhibitors (ICIs) have emerged as novel choices, their clinical response rates are rather limited. To date there is a lack of effective treatment regimens for patients with metastatic or recurrent cervical cancer. Recently nanomaterials like liposomes, dendrimers, and polymers are considered as promising delivery carriers with advantages of tumor‐specific administration, reduced toxicity, and improved biocompatibility. Here, we review the applications of nanoparticles in the fields of drug delivery, CRISPR based genome‐editing and therapeutic vaccines in cervical cancer treatment.

This article is categorized under:

Therapeutic Approaches and Drug Discovery > Nanomedicine for Oncologic Disease

## Graphical abstract

Recent progresses in nanoparticle‐based delivery systems show potential foreground for enhancing clinical benefits while minimizing systemic toxicities in cervical cancer.

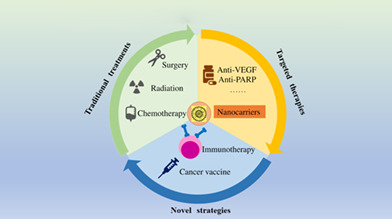

## 1 INTRODUCTION

Cervical cancer has been the second leading cause of cancer deaths among females with over 500,000 new cases a year (Liu et al., 2019). In general, cervical cancer can be classified as squamous cell carcinoma, adenocarcinoma, and adenosquamous carcinoma according to the histology (Lavanya et al., 2020). Infection with human papillomavirus (HPV) has been confirmed to be a key prerequisite for the 90% of cervical cancer (Marshall et al., 2018). In addition, HPV‐16 and HPV‐18 are main driven factors for the malignant transformation of cervical epithelial cells (Shibata et al., 2019). Recent investigations show that HPV‐16 is responsible for almost 80% of cervical cancer, which belongs to squamous cell carcinoma while HPV‐18 is related to 20% of cervical cancer that diagnosed as adenocarcinoma (Anderson et al., 2001).

For cervical cancer, multiple strategies such as surgery, chemoradiotherapy, and adjuvant therapies like targeted therapy and immunotherapy are recommended as the standard treatment (Cohen et al., 2019). In considering the strategy that surgery treatment is applied to cervical cancer patients only in very early stage(IA‐IIA), thus radiotherapy or concurrent chemoradiotherapy account for the major treatment of locally advanced (IIB‐IVA) or recurrent sufferers. To date, the treatment efficiency of cervical cancer remain unsatisfactory, more than 30% of the initial treated patients was diagnosed as relapse and metastasis within 2 years, and the 5‐year survival rate was less than 10% (Li et al., 2018). For the patients with recurrence or metastasis, cisplatin, and paclitaxel drugs‐based chemotherapy together with tumor vascular‐targeting drugs such as bevacizumab (targeting vascular endothelial growth factor, VEGF) have been approved by the food and drug administration (FDA) as the first‐line treatment according to the results of GOG 240 trial (Bradley, & Krishnansu, 2014). However, cisplatin‐based chemotherapy often leads to drug resistance, while new treatment methods such as VEGF targeting therapy and immunotherapy have shown limited effect. For instance, the overall responses rate (ORR) of pembrolizumab (PD‐1 inhibitor) in total population was merely 12.2% in KEYNOTE‐158 trial (Chung et al., 2019). Therefore, developing novel strategies for the improvement of treatment efficiency of cervical cancer is urgently needed.

As mentioned above, traditional regimens are nonspecific and limited, the cytotoxicity along with drug resistance are two main challenges affecting clinical decisions and prognosis of patients (Sharma et al., 2020). In view of this, nanocarriers are expected to increase the sensitivity of chemoradiotherapy and reduce systemic toxicities through targeted administration and enhanced permeability/retention effect (EPR; Guo et al., 2019). The implication of EPR effect is that nanocarriers preferentially aggregate in tumor sites rather than normal sites because of the poor tissue integrity, wide vessel wall space and abundant blood supply in solid tumors (Garbayo et al., 2020). Thus, nano‐vehicles possess the superiorities of targeted administration, reduced systemic toxicity and lower degradation rate (Rajithaa et al., 2019). In the past few years, nanoparticle‐based applications have presented great clinical transformation potential when combined with drug delivery, targeted gene editing, and therapeutic cancer vaccines in cervical cancer.

## 2 DRUG DELIVERY FOR CERVICAL CANER VIA NANOMATERIALS

**FIGURE 1:**
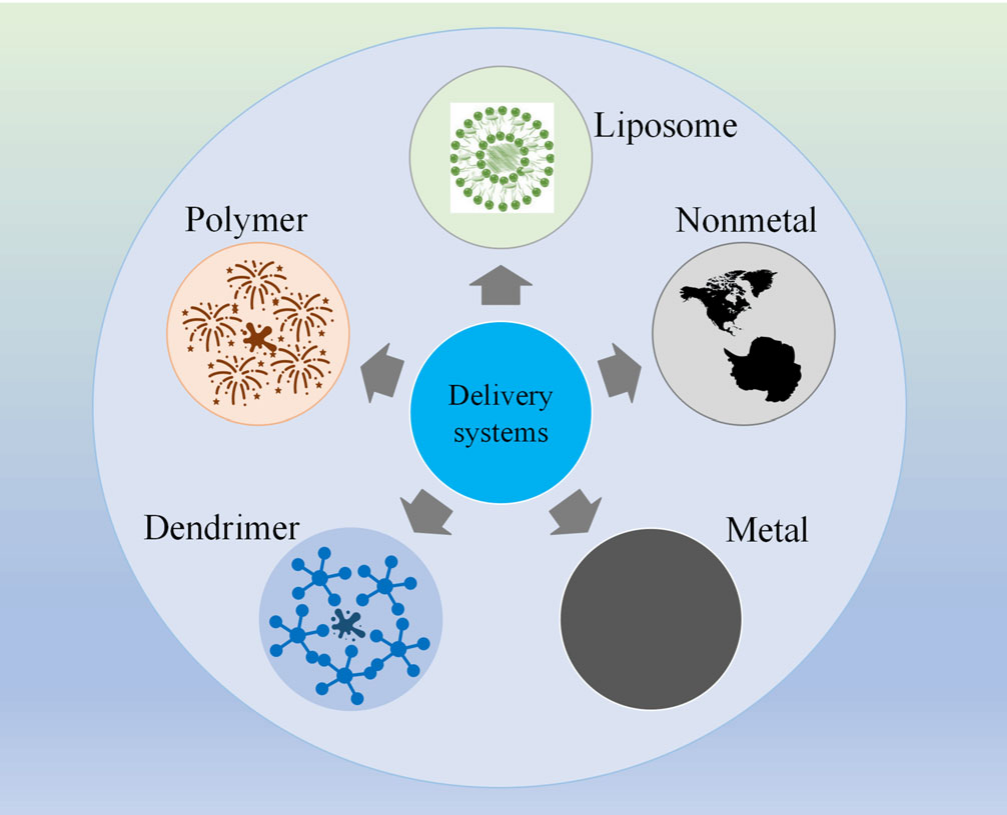
Various delivery systems applied in cervical cancer treatment

Nanocarrier‐based delivery systems have been extensively investigated in recent years, due to the promotion of sustained drug release, systemic distribution, and reliable absorption efficiency (Pathak & Akhtar, 2018). These vehicles are conducive to drug administration and can be classified as liposomes, polymers, dendrimers, and inorganic materials(metallic or nonmetallic), and so on (as shown in Figure 1).

### 2.1 Liposome‐based nanoparticles

Amphoteric liposomes can overcome membrane‐mediated barriers for the delivery of chemotherapeutic drugs owing to their flexible properties like zeta potential, particle diameter, and controlled‐release ratio (Smerkova et al., 2019). The lipid‐carriers have superiorities in regard of improving drug penetration and minimizing systemic administration time through lipophilicity/hydrophilicity and EPR effect (Garbayo et al., 2020).

**FIGURE 2:**
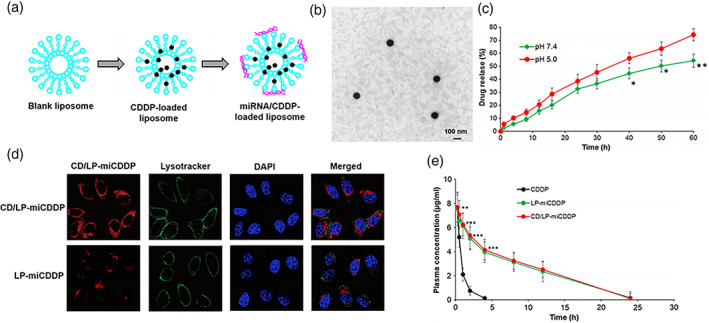
Overview of experimental pathway for CDDP and miRNA loading. (a) The CDDP was loaded in the liposome shell while miRNA on the surface. (b) Morphology detection of CD/LP‐miCDDP with transmission electron microscopy. (c) Drug release study of CD/LP‐miCDDP in Ph 7.4 and Ph 5.0 conditions. (d) Cellular uptake analysis of CD/LP‐miCDDP and LP‐miCDDP in cervical cancer cells. (e) Plasma concentration–time analysis of CDDP, LP‐miCDDP and CD/LP‐miCDDP. *p <0.05, **p <0.01, ***p <0.0001. Copyright © 2020 SpringerOpen

Cisplatin is an effective first‐line chemotherapeutic drug for cervical cancer as described before (Liontos et al., 2019). To reduce drug resistance and toxicity, Dana et al. (2020) developed one cisplatin‐related carrier based on liposomes and poly lactic‐co‐glycolic acids (PLGA) through double emulsion solvent evaporation approach, the anti‐angiogenic drug Avastin was also conjugated to the lipid system (L‐PLGA‐Cis‐Avastin). The results of 3D spheroid and xenograft experiments showed that L‐PLGA‐Cis‐Avastin system had better cellular uptake potential and greater binding capability. Another group designed a kind of conjugate combined CD59, miRNA‐1284 with cisplatin (CDDP), and then loaded them with liposomes (CD/LP‐miCDDP). This co‐delivery system showed better anticancer effects in cervical cancer cells, and apoptosis rate was significantly increased compared to cisplatin or miR‐1284 alone (60 vs. 20% and 12%, respectively). What is more, the maintenance of encapsulated drug in blood circulation revealed a 6.9‐fold higher than that of in cisplatin group, while the clearance rate had an 8‐fold decrease (as shown in Figure 2; Wang & Liang, 2020). In addition, the combination of prodrugs 2T–P and 2T–N (conjugates of phospholipid tails (2T), podophyllotoxin (P), and the analogue (N)) with lipidic components could increase drug stability and incorporation in cervical cancer cells, and hence was beneficial for local administration (Márquez et al., 2020). Therefore, lipidic vectors can improve cellular uptake and reduce side effects of cisplatin both systematically and locally.

Photodynamic therapy (PDT) is a new anti‐cancer strategy and has gained widely concern in the past few years. PDT is photosensitizers dependent and can release reactive oxygen species (ROS) as well as free radicals in the presence of appropriate absorption wavelength (Fang et al., 2020). To improve the efficiency of drug delivery, Singha et al. (2018) designed the dihydroindolizine (DHI) encapsulated lipid nanoparticles. DHI is one class of photochromic material that can be light‐induced to produce pyrroline ring opening and recovery reactions of thermal back. Based on these characteristics, the ammonium salt of 8‐anilino‐1‐naphthalenesulfonic acid was incorporated in lipid materials. In this study, the stability of liposomal membrane was affected in pace with structural changes of DHI (from closed isomer to open isomer). Due to the lipid‐based photo‐responsively reversible nature, the uptake of doxorubicin (DOX) was significantly enhanced and the viability had a 40% reduction in cervical cancer cells. Thus, liposome‐photosensitive vehicles can be applied on the delivery of chemotherapeutic drugs as well.

### 2.2 Polymeric nanoparticles

Polymeric nanoparticles (PNPs) are composed of polymers with core‐shell such as polysulfide‐ester, polyacrylate, and gelatine with the diameters ranged from tens to hundreds of nanometers. PNPs had been used as drug carriers for cervical cancer owing to the characteristics of flexible controlled‐release, TME (tumor microenvironment) responsive, and improved solubility of insoluble anti‐cancer drugs (Garbayo et al., 2020).

In a recent study, the Poly (DEA)‐b‐Poly (ABMA‐co‐OEGMA) (referred to as PDPAO) was grafted with prodrug of reduction‐dependent 6‐mercaptopurine (6MP) and pH‐responsive DOX. After characterizing, this pH and reduction sensitive polymers (average particle size was 116 ± 2 nm) had firm sphere structures and small dispersion index. What is more, this subtle drug release system could be triggered by the double switch‐pH/GSH in acidic tumor environment. As expected, the controllable polymers presented remarkably increased cellular uptake and enhanced cell‐killing capacity in cervical cancer cells (Liao et al., 2020). In another study, the polymeric prodrugs based on hyaluronan and DOX were developed in a self‐assembly manner, with the features of pH‐dependent release and repeatable stability in vitro. These polymeric vehicles exhibited higher cytotoxicity and apoptotic ratio exclusively in HeLa cells (Liao et al., 2018). The pH‐dependent polymeric release system could also reduce dose‐related toxicity of anticancer agents‐chlorambucil (CBL). For example, a pH‐sensitively autofluorescent polymeric nanoplatform was established to promote the uptake of CBL. The luminescent copolymer conjugating with PEG‐styrene‐CBL complexes were self‐assembled into 25–68 nm particles in which the CBL was centrally encapsulated. Under the condition of pH 5.0, the CBL‐mediated release ratio was up to 90% via cleavage of aliphatic ester (linkages between CBL and maleimide units). Besides, the CBL‐polymer presented more cellular internalization and greater cytotoxicity in cervical cancer cells than that of in normal cells (Saha et al., 2019). The pH‐responsive polymers may represent available approaches to deliver drugs intracellularly.

In addition to conventional drugs, immunomodulators encapsulated with PNPs could also display therapeutic potential in HPV‐related cancers (Yang et al., 2014). Silver‐polymeric nanocarriers composed of chitosan‐graft‐poly and polyethylene glycol (PEG) increased tumor‐specific toxicity at the concentration of 8 μg/ml in HeLa cells (Banerjee et al., 2016). In addition, as an activator of toll‐like receptor 7 (TLR7), imiquimod can both activate innate immune cells and promote the expression of inflammatory factors such as IFN‐α and IL6,8,12 (Hengge & Ruzicka, 2004). PNPs‐imiquimod obviously inhibited cell viability and induced 80% apoptosis of SiHa cells by stimulating the secretion of IL‐6 (Franka et al., 2019).

### 2.3 Dendrimer conjugates as nanocarriers

Dendrimers have been extensively investigated as delivery carriers because of their special branching structure with cavities and core inside which can form nanocarriers of precise molecular structure and high geometric symmetry. Dendrimers contain abundant functional groups on the surface that can either conjugate with small molecules via hydrophobic and electrostatic interaction, or be modified with diverse small‐molecule drugs (Aggarwal et al., 2018). To minimize the cytotoxicity of surface groups on dendrimers, a new kind of nontoxic surface coating was developed. The special surface was composed of 6‐hydroxyhexanoyl/oxy‐hexanamide and phosphoryl choline hexanamide. The specially optimized dendrimers presented significantly lower toxicities than conventional PAMAM‐dendrimers in cervical cancer cells (Svenningsen et al., 2016).

Small molecule chemotherapeutic drugs have several shortcomings including poor bioavailability, low responses rate to treatment and serious side effects (Liontos et al., 2019). In consideration of this, ligand guided dendrimers may be an alternative choice to deliver drugs to cancer cells over‐expressed with specific receptors. Luong et al. (2016) successfully increased the aqueous solubility of flavonoids analogues for cancer with folate modified dendrimers. Interestingly, the targeted agents showed more intracellular accumulation and increased apoptotic proportion of HeLa cells. Chlorin e6 (Ce6) is a potential candidate in PDT because of its strong phototoxicity and light absorbing capacity (He et al., 2016). To overcome the drawbacks of poor water solubility and undesirable cell localization of Ce6, Lee et al designed a novel hydrophilic nano‐copolymer (DC) with poly‐amidoamine dendrimers. The spherical DC with an average particle size of 61.7 nm displayed not only better cellular uptake but also stronger cytotoxicity than Ce6 alone (Lee & Kim, 2018).

### 2.4 Inorganic nanocarriers

Inorganic nanocarriers can be classified as nonmetallic materials including graphene oxide (GO), silica or carbon, and metallic vehicles such as gold and copper. Diverse inorganic materials have been used as gene or drug vectors with the properties of more effective cellular uptake and higher dispersibility (Paris et al., 2019). However, the feature of low degradation rates and potential toxicity are also non‐negligible problems, and structural optimization is necessary to ensure the internal degradation and clearance in a secure way.

GO is a class of single‐atomic‐layered material made of an array of carbon oxide atoms. GO materials have been used in many fields like gene delivery and cancer vaccine (Cao et al., 2020; Daniyal et al., 2020). Primary GO is not suitable for gene delivery because of the difficulty in loading dsDNA. For this reason, the GO nano‐vehicles coated with cationic liposome 1,2‐dioleoyl‐3‐trimethylammonium‐propane (DOTAP) (GOCL) were designed. Through characterizing the size and surface charge, GOCL nanocarriers showed appropriate physical and chemical properties for DNA payload (Santo et al., 2019). Silica vectors can also act as drug reservoirs. Duo et al recently enveloped CX‐5461 (an inhibitor specially targeting RNA polymerase and inducing tumor cell autophagy) with mesoporous silica nanoparticles (referred to as MSNs), and then loaded the latter with polydopamine (PDA), PEG and AS‐1411. In this system, PDA could avoid the seepage of CX‐5461, PEG improved the stability and biocompatibility and AS‐1411 increased nucleolar aggregation. The size, potential and encapsulation of this nanoplatform were characterized, without obvious toxicity on viscera of mice. As expected, reconstructed MSNs‐CX‐5461 not only showed higher cytotoxicity, but exhibited better distribution and growth inhibition in Hela xenografts models (Duo et al., 2018). Another group obtained stable calcium carbonate nanoparticles (CCNs) in an ethanol dehydration approach, with human serum albumin (HSA) conjunct on the surface. The HAS was then conjugated with naked CaCO_3_ and CaCO_3_‐NH_2_ (modified with aminopropyl triethoxysilane), respectively. Effective internalization of the two nano‐vehicles in HeLa cells was both confirmed by confocal microscopy, and the CCNs‐HAS groups showed distinctly increased cellular uptake compared with naked CCNs. The efficiency of cellular uptake was affected by surface charge of CCNs (Vergaro et al., 2019).

Metallic NPs have been widely applied to initiation of immunoreactions, gene delivery, and radiosensitivity enhancement (Alphandéry, 2020; Kratošová et al., 2019). A current study presented that spherical chitosan coated oxide nanoparticles (CuONPs@CS) could activate macrophages and initiate robust anti‐tumor immunoreactions. Toxicological experiments showed that chitosan promoted the release of cuions and killing of cervical cancer cells without distinct lymphocytes toxicity at dose of 50 μg/mL. The metal conjugates markedly suppressed proliferation of HeLa cells in vitro and growth of tumor in Balb/C mice through secretion of cytokines (TNF‐α, IFN‐γ, and IL‐12) as well as activation of Th1/2 cells (Deya et al., 2020). Gold nanoparticles (AuNPs) have also become available carriers for drug administration (siRNA, DNA, or proteins) owing to the properties of high bioavailability, great surface area, and flexible dispersion (Matteis et al., 2019). To deliver small interfering RNA (siRNA) into tumor sites, Yi et al. (2016) developed a targeted AuNPs, a class of monodispersed unimer polyion complex (uPIC) that was composed of therapeutic siRNA, cyclic RGD, and block catiomer, the assembled uPIC was then modified onto AuNP (20 nm). This nanocarrier system not only delivered siRNA that targeted HPV E6 gene efficiently, but inhibited xenograft tumors, which derived from HeLa cells specifically. In addition, increasing evidences showed that metallic NPs could improve radiosensitivity and attenuate systemic toxicity at the same time (Martinez et al., 2020; Pagáčová et al., 2019). Yang et al. (2020) developed one biocompatible material PEGylated platinum nanoflowers (Pt NFs). Through integrating with radiation‐induced molecular and grafted polymers, Pt NFs located in cytoplasm of HeLa cells, and distinctly promoted radiotherapy induced apoptosis with 23% increase of sensitizing enhancement ratio in HeLa cells. In addition, these Pt NFs could be surface modified with molecules such as radionuclides, enzymes or fluorescein. Besides, synthesized AuNPs could act as radiosensitizers and accelerated the cytotoxicity of HeLa cells by 9.93 times compared to radiotherapy alone (Shanei & Zadeh, 2019).

### 2.5 Multifunctional nanoparticles

Up to present, the clinical application of nanoparticle‐based drug administration is still limited. The main reason is that single nano‐platform mentioned above can only overcome part of the obstacles during drug delivery in vivo such as limited targeting, poor biocompatibility and short circulation time (as shown in Table 1 **)**. The development of multifunctional NPs is one of the effective way to address these barriers. Multifunctional NPs composed of two or more different NPs with similar or dissimilar compositions, and have been applied in multiple anti‐cancer fields including delivery of chemotherapeutic drugs, induction of cancer cell apoptosis and synergize with PDT (Habibi et al., 2020).

**TABLE 1:** The characteristics, limitations, and applications of different nanoparticles (NPs)

Types of NPs	Components	Characteristics	Limitations	Applications
Liposome‐based NPs	Solid core lipid with bilayer structure	1. Good biocompatibilities for both hydrophilic and hydrophobic drugs; 2. Stable structure; 3. Easy to be surface modified.	The tendency of drug release before reaching the target site in vivo due to poor physical and chemical stability	Cisplatin‐related drug delivery with lipid nanomaterials to reduce drug resistance and toxicity (Márquez et al., 2020)
Polymeric NPs	Polymers based core‐shell NPs	1. Improved solubility of insoluble anti‐cancer drugs; 2. Better drug loading capacity; 3. Controllable drug release.	1. Poor drug permeability; 2. Drug bioavailability needs to be improved.	pH‐responsive doxorubicin loading with polymeric NPs to enhance drug uptake (Liao et al., 2020)
Denrimer‐based NPs	A large number of branching units with cavities and core inside	1. Precise molecular structure; 2. High geometric symmetry; 3. Abundant functional groups on the surface.	1. Absence of definite mechanism; 2. Lack of definite and in‐depth research on mechanism of chemical reactions.	Promotion of Chlorin‐e6 in PDT with dendrimer NPs platform (He et al., 2016)
Inorganic NPs	nonmetallic materials (graphene oxidesilica or carbon) or metallic NPs (gold and copper)	1. Stable structure; 2. Larger specific surface area; 3. Easy surface functionalization.	Potential toxicity owing to the low degradation and clearance rates in vivo.	Delivery of siRNA into tumor sites with Gold nanoparticles (Matteis et al., 2019)

Chemothermal therapy is a novel strategy for cervical cancer treatment. To enhance tumor killing efficiency, one kind of multifunctional FePt‐Fe_3_O_4_ NPs (CNAs) were developed. The multifunctional NPs were highly aqueous stable and carboxyl enriched, and presented high loading ability (90%). CNAs loading with DOX exhibited pH responsive release capacity, what is more, drug release could be enhanced by alternating current magnetic field. CNAs were able to produce ROS with the help of Fe and Pt in the NPs and hydrogen peroxide in cancer cells. This multifunctional nano‐platform specifically killed tumor cells but has no effect on normal cells by taking advantage of DOX delivery and ROS generation (Sahu et al., 2015).

Induction of cell apoptosis is another application of multifunctional NPs in cervical cancer. You et al recently synthesized multifunctional nano‐noisome through loading anti‐cancer drugs curcumin and folic acid in the Fe_3_O_4_@PLGA‐PEG drug delivery system. This NPs above possessed several features: (a) excellent biocompatibility, (b) good drug carrier capacity, (c) significantly high targeting efficiency in vitro. And better yet, the niosomes loading with curcumin dramatically induced HeLa229 cell apoptosis via changing mitochondrial membrane potential and destroying cell cycle (You et al., 2019). The ursolic acid (UA) liposome coated with chitosan could also promote cell apoptosis of cervical cancer. With the help of chitosan modification, positively charged liposomes tended to interact with negative charged tumor cells. In addition, UA could be released rapidly in acidic TME. The CS‐UA‐L treated U14 tumor bearing mice had significantly enhanced necrosis and cell apoptosis (Wang et al., 2017).

Multifunctional NPs also played crucial roles in PDT for cervical cancer. Zheng et al. (2019) designed one Au@TiO2 core‐shell NPs which could take advantage of broad photoabsorption and enhance ROS generation. They first loaded the Au@TiO2 core‐shell NPs with chemotherapeutic drug DOX, and then a zwitterionic and pH responsive polymer was applied to improve the biocompatibility and prolong the circulation halftime. Notably, Au@TiO2 NPs could produce enhanced contrast image under T1/T2‐weighted magnetic resonance imaging (MRI) through chelating with Mn^2+^. In line with expectations, this multifunctional nano‐platform displayed significantly significant tumor killing efficiency and minimal side effects.

## 3 NANO‐CRISPR SYSTEM FOR CERVICAL CANCER TREATMENT

In recent years, the combination of nanoparticles and emerging genome‐editing methods such as Clustered regularly interspaced short palindromic repeats (CRISPR) has triggered an innovation in targeted therapy for cervical cancer. Deriving from the adaptive immune defense system of bacteria and archaea, CRISPR system contains Cas9 endonuclease and single‐guide RNA (sgRNA), the sgRNA can guide Cas9 moving forward and then targeting specific sequences (Chong et al., 2020; Wada et al., 2020). CRISPR‐dependent editing processes can be realized through three systems: gRNA ribonucleoprotein (RNP), plasmid, and mRNA system (Jiang et al., 2019). The most often used RNP system can simplify editing steps without transcription and translation, but may cause contamination of endotoxin and reduced delivery efficiency. Plasmid system is more stable and economic compared to RNP system, nevertheless, its large size is a non‐negligible obstacle for plasmid delivery and targeting. mRNA system is an alternative method in regard to lessening off‐target ratio, but it is inherently unstable (Knott & Doudna, 2018; Wang, Zhang, et al., 2020).

Though both viral and physical delivery methods had been validated in reducing degradation and ensuring precise targeting, the applications of viral vectors were restricted due to its strong immunogenicity, increased risk of certain cancer and complicated processes. On the contrary, nonviral delivery systems like inorganic and lipidic nano‐vehicles are showing great foreground (Filipczak, Pan, Yalamarty, & Torchilin, 2020). For example, AuNPs have special physicochemical characters for targeted delivery as described before (Matteis et al., 2019). Ju et al. (2019) developed a pH‐dependent and self‐assembled AuNPs to deliver Cas‐9 into nucleus, and then to knockout E6 gene as well as restore the expression level of p53. In addition, another self‐assembled micelle composed of polyepoxypropane and amphiphilic‐pluronic was established to encapsulate the plasmid CRISPR system. As expected, micelle‐E7‐Cas‐9 system completely induced E7 knockout and remarkably suppressed cell viability in xenograft model of HeLa cells (Lao et al., 2018).

**FIGURE 3:**
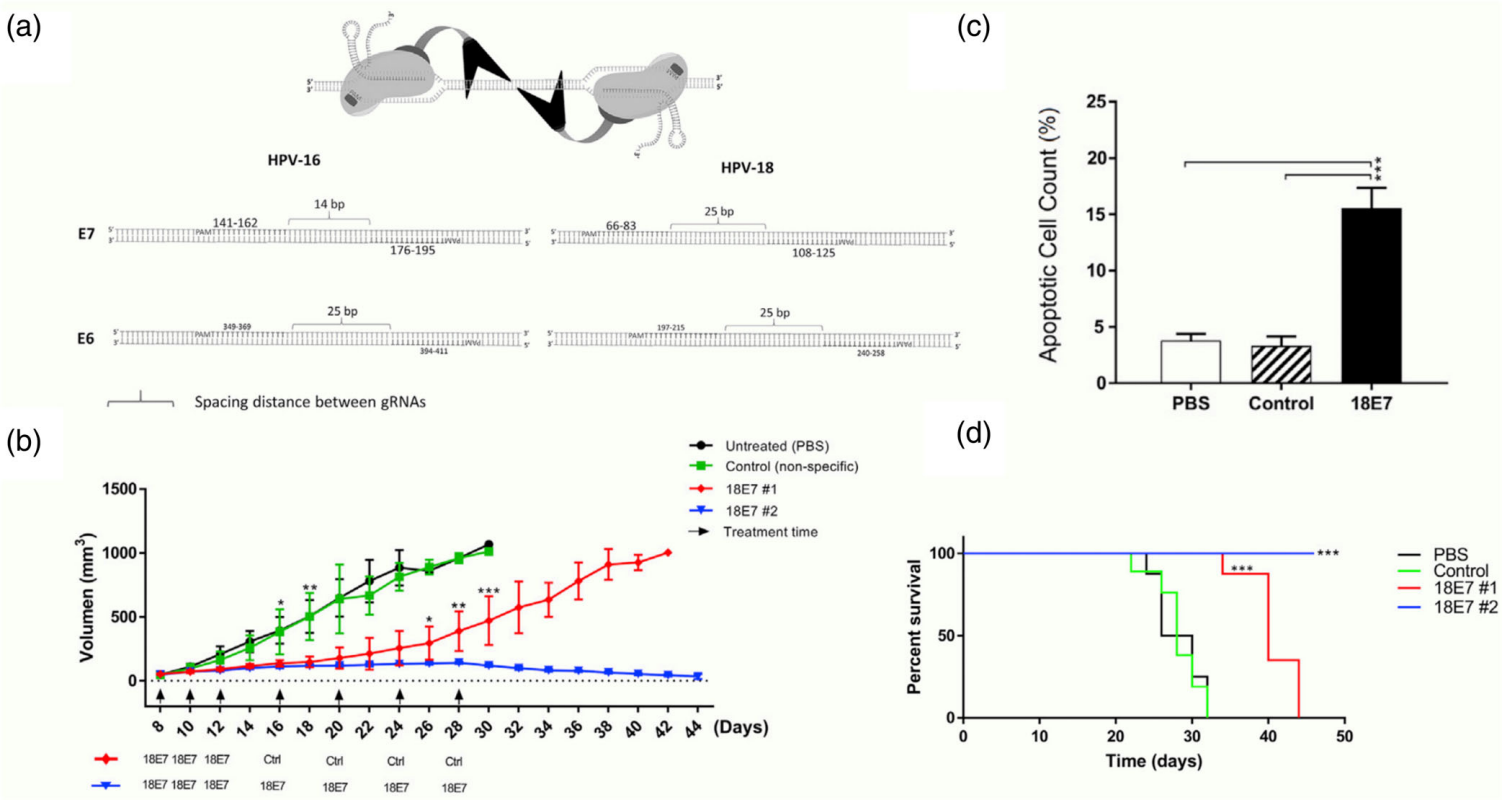
Systemic administration of lipid‐CRISPR system cleared solid tumors through apoptotic pathway. (a) Binding sites for gRNAs on E6 and E7 genes of HPV. (b) gRNAs (18E7) + Cas9‐lipid effectively eliminated xenografts tumors derived from HeLa cells. (c) The apoptosis ratio was increased in 18E7 group. (d) The lipid‐CRISPR system prolonged mice survival in 18E7 group. Open achieved. Copyright © 2019 Elsevier B.V

Liposomes can provide platforms for nonviral genome editing as well. Recently a self‐assembled cationic lipid NPs (CL‐NPs) was successfully established to deliver CRISPR. This complex was pH‐responsive with high efficiency of gene knockout and tumor‐specific targeting. CL‐NPs distinctly restrained cell survival and promoted cell apoptosis of cervical cancer by inactivating E6/E7 genes in vitro, and furtherly suppressed the growth of HPV‐positive tumor (Zhen et al., 2019). Liu et al. (2016) displayed that the lipofectamine coated CRISPR system could specifically suppress the growth of E7‐transfromed cells. Further, the first case using stealth liposomes for the delivery of CRISPR system in cervical xenografted tumor was reported recently. Jubair et al. (2019) confirmed that EGylated lipid‐carriers are both safe and available in vivo. gRNAs and Cas9 endonuclease were encapsulated in PEGylated lipid complex (LCas‐E6/E7) with average size 210 nm and zeta‐potential +45 mV. PEGylated lipid was able to prevent plasmid DNA from degradation up to 6 h in serum. The LCas‐E6/E7 system remarkably inhibited tumor growth and promoted cell apoptosis through increasing the expression of activated caspase‐3 protein. Besides, through confirming of p16 IHC staining, the remnants of tumor regression were replaced by stromal cells (as shown in Figure 3). Hence, the lipid‐CRISPR platforms are effective for genome editing in vivo. However, the critical elements need to be considered are that CRISPR–Cas9 may bring about insecure results such as chromosome abnormalities and nontarget genome cleavages in human (Knott & Doudna, 2018; Wang, Zhang, et al., 2020).

To address the questions above and understand the safety and feasibility of CRISPR editing in clinical application, Lu et al. (2020) conducted the first phase I clinical trial of PD‐1‐T cells using CRISPR technology in patients with metastatic nonsmall‐cell lung cancer. The median OS and mPFS was 42.6 and 7.7 weeks, respectively in 12 recipients, and 12 weeks after PD‐1‐T cells infusion, obviously tumor regression was witnessed. And better yet, the median off‐target mutation rate was merely 0.05% via whole‐genome sequencing (WGS) and next generation sequencing (NGS) detection. Thus, CRISPR‐T cells strategy was reliable and the off‐target events were fairly limited in this trial. Considering the insufficiency of targeting efficiency and bioavailability in this research, the combination of nanocarriers and CRISPR may be a prospective approach for targeted gene therapy in cervical cancer in the near future.

## 4 NANOPARTICLE‐BASED THERAPEUTIC VACCINES FOR CERVICAL CANCER THERAPY

### 4.1 Components of therapeutic cancer vaccines

Although prophylactic vaccines against cervical cancer have been widely used, they are unable to eliminate previously existing tumor cells tumors. Therefore, it is critical to develop therapeutic vaccine against cervical cancer. Unlike prophylactic vaccines that produce neutralizing antibodies against pathogens, therapeutic cancer vaccines are designed to induce durable and powerful immune reactions through delivering tumor‐specific antigens to lymphatic system and then activating antigen presenting cells (APCs; Maeng et al., 2018). In general, therapeutic cancer vaccines embrace four crucial components: cancer antigens, diverse formulations, adjuvants, and delivery carriers. Tumor antigens are most likely the top priority to be identified and can be classified as tumor associated antigens and tumor specific antigens (Hu et al., 2018). Vaccine formulations mostly compose of all antigens (whole cancer cells) and specific antigens such as proteins, multi‐peptides, or nucleic acid (DNA or mRNA; Hu et al., 2018). As antigen itself is difficult to trigger potent immune reactions, adjuvants such as TLR agonists and stimulators of interferon genes (STING) are of crucial importance in activating adaptive immune responses (Elsas et al., 2020; Wang et al., 2020). Further to the above, suitable vaccine delivery vectors are essential prerequisite to bring antigens to lymph nodes and prevent them from degradation (Hu et al., 2018). Despite responses induced by cancer vaccines are durable, the tolerability and efficiency are two grand challenges. Nanomaterials including liposomes or polymers have emerged as desired vehicles to develop vaccines that can produce robust antigen‐specific reactions of perfect safety and immunogenicity (Medina‐Alarcón et al., 2017).

### 4.2 Nanocarriers in therapeutic vaccines of cervical cancer

Therapeutic vaccines against HPV antigens can be classified as DNA/mRNA, proteins/polypeptides, vectors (bacterial or viral), dendritic cells (DCs), and adoptive cells (as shown in Table 2; Clark & Trimble, 2020). DNA therapeutic vaccine is the best studied type among them in cervical cancer. A phase IIb study showed that women with cervical intraepithelial neoplasia 2/3 (CIN2/3) could be treated by VGX‐3100, the first effective DNA vaccine targeting both E6 and E7 fragments of HPV16/18, and the ratio of histopathological regression had nearly 19% increase in VGX‐3100 compared to that of in placebo (49.5% in VGX‐3100 vs. 30.6% in placebo; Trimble et al., 2015). Despite the positive effects in precancers, naked DNA vaccines have not displayed sufficient efficacy in existing cervical cancer. The possible mechanisms could be partially interpreted as low bioavailability, DNA degradation and poor clinical responses that caused by increased expression of suppressive immunosuppressive regulatory T cells (Tregs) or PD‐1/PD‐L1 (Schreiber et al., 2011; Sica & Massarotti, 2017).

**TABLE 2:** Characteristics of human papillomavirus therapeutic vaccines

Types of vaccines	Antigens	Advantages	Disadvantages
DNA	E5; E6; E7	Good stability; Easy to build	Low immunogenicity; Increased cancer risk
mRNA	E7	Genomic non intergrating; Multiple antigens encoding; Cost‐effective	Poor stability; Ineffective delivery
Protein	E6; E7	High security; HLA unrestricted	Excessive cytotoxicity
Polypeptide	E6; E7	Easy to produce; High purity	Specially designed peptides
Vector	E2; E6; E7	Large fragments availability; Adjuvant function	Low immunogenicity; Decreased safety
Dendritic cell	E7	Specific antigen presentation and T cell immuneresponse	Specific HLA type of tumor antigens
Adoptive cell	E6; E7	Selective antitumor immunity	Lack of high affinity T cells of tumor antigen specific

To overcome the obstacles above, adjuvants and vehicles of nanoscale were attempted to enhance cytotoxicity of therapeutic vaccines against cervical cancer. For instance, the combination of adjuvant CpG/GPI‐0100 with TVGV‐1 (HPV16 E7 and pseudomonas exotoxin‐based vaccine) could contribute to forceful immunoreactions. CpG‐TVGV‐1 conjugates induced secretion of IFNc and enhanced high immunogenicity (Silva et al., 2019). Similarly, the addition of HPV peptide vaccine with adjuvants (like CpG and α‐GalCer) also induced higher efficiency of cytotoxic CD8^+^ T cells and decreased the proportion of Tregs and myeloid derived suppressor cells (MDSCs) in HPV‐driven mice (Sierra et al., 2020). Another group found that nano‐adjuvant TLR 9 system together with HPV16‐E7 promoted the infiltration of MDSCs, raised the number of local CD8^+^ T cells and prolonged mice survival (Pereira et al., 2019). The additional components of IL‐12 and nanocarrier chitosan to E7 vaccine both gave rise to higher expression of cytokines (IFN‐γ, IL‐4) and produced more obvious suppression of tumor growth than HPV‐E7 vaccines alone (Tahamtan et al., 2018). Besides, Human telomerase reverse transcriptase (hTERT) can induce immortalization of tumor cells by maintaining the telomere length, and may be a suitable target for treatment (Simone Negrini et al., 2020). A chitosan encapsulated PLGA‐nanocarriers were designed to deliver siRNA and target hTERT mRNA. The results presented that the siRNA‐chitosan‐PLGA based NPs could distinctly accelerate apoptosis of HeLa cells in the manner of high efficiency and extended release (Nagapoosanam et al., 2019). In addition, Archaeosomes originating from biological liposomes of archaea have the power to stimulate and recruit DCs for a potent antigen presentation (Schwendener, 2014). In a recent study, truncated fragments L1/E6/E7 of HPV16 genes were developed for tumor antigens, in the meantime archaeosomes was double used as adjuvant and nanocarrier. As expected, the conjunction of archaeosomes and L1/E6/E7 presented greater cytolytic capability and therapeutic potential in mouse model (Karimi et al., 2020).

Due to negative characteristics of DNA, the cationic liposomes are considered as attractive vectors for DNA cancer vaccines. PDS0101, the lipidic HPV vaccine consisted of HPV16 peptides and cationic lipid (R‐DOTAP), could promote the infiltration of CD8^+^ T cells (Jochems et al., 2017). Rumfield et al. (2020) recently added immunomodulators M7824 and NHSIL12 to the PDS0101. M7824 was made up of extracellular fragments of transforming growth factor receptor (TGFβR) and then fused with PD‐L1, thus it could both act as checkpoint inhibitor and bring TGFβR to the TME. Similarly, NHSIL12 was designed to bring IL‐12 to the TME and promote Th1 related inflammatory responses. Surprisingly, the combination of PDS0101 and two immunomodulators showed obviously increased clonal expansion ability of T cells as well as powerful anticancer effect. In addition, polymeric nano‐vectors were conjugated to HPV‐E7 long peptide(E7LP) by Galliverti et al. (2018). The NP‐E7LP led to the accumulation of CD8^+^ cytotoxic T cells without gathering Tregs and thus produced more intense therapeutic efficacy.

**FIGURE 4:**
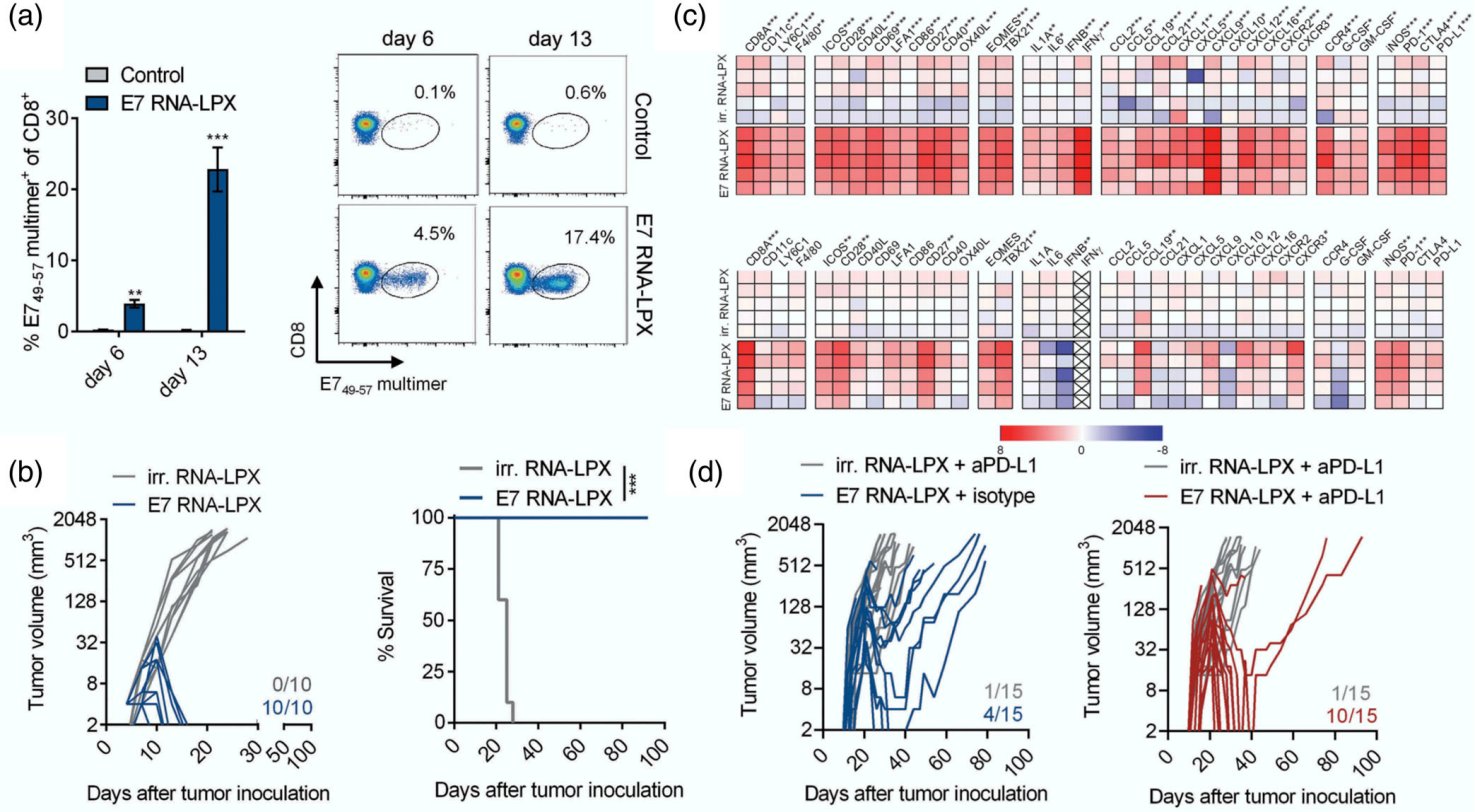
Lipid‐mRNA vaccine mediated significantly efficacy in HPV‐positive tumors. E7‐lipid‐mRNA vaccine promoted the infiltration of memory CD8+ T lymphocytes (a), complete remission of HPV16‐positive tumors (b), secretion of proinflammatory factors (c), and inhibition of anti‐PD‐L1 refractory tumors (d). (Reprinted with permission from Grunwitz et al. (2019). Copyright © 2020 Informa UK Limited

Compared to the evolution of DNA vaccines, the development of mRNA vaccines was lagging behind mainly because of the ineffective delivery and inherent instability in vivo. Recently, mRNA vaccines have shown great foreground for treatment with the progresses of new delivery materials like cationic liposomes and optimized preparation processes (Pardi et al., 2018). The mRNA vaccines have obvious superiorities over other vaccines including genomic nonintegrating, low vector‐related immunogenicity, multiple tumor antigens encoding simultaneously, and cost‐effective productions (Pardi et al., 2020). What is particularly noteworthy is that mRNA therapeutic vaccine has been demonstrated effectively in a phase I clinical trial just recently. Sahin et al. (2020) designed a liposomes‐based mRNA vaccine with four tumor‐associated antigens (NY‐ESO‐1, MAGE‐A3, TPTE, and tyrosinase) of melanoma (FixVac). FixVac alone or together with ICIs could both activate durable and potent cytotoxic CD8^+^ T‐cell and CD4^+^ T‐cell immune reactions. Cationic lipid‐mRNA had also been established in cervical cancer cells. With the capacity of enhancing mRNA stability, vectors pST1‐A120 and pST1‐MITD were used as the templates for HPV antigen‐encoding mRNA. E7 protein was encoded by pST1‐E7‐MITD plasmid system through cloning sequences, and the liposome vectors were composed of cationic lipid DOTMA and helper DOPE (Kranz et al., 2016; Kreiter et al., 2007). The established liposome preparation was named as RNA‐LPX with average particle size 200–250 nm and appropriate zeta potential (−20 to 30 mV). The targeting mRNA sequences were encapsulated in RNA‐LPX and then presented by DCs in mouse models. In line with expectations, the E7‐RNA‐LPX triggered significantly proliferation of cytotoxic lymphocytes and complete remission (CR) of HPV positive tumors. What is more, through inducing robust CD8^+^ T cell responses, E7‐RNA‐LPX could synergize with ICIs to reverse immunotherapeutic resistance in anti‐PD‐L1 refractory tumors (Grunwitz et al., 2019; as shown in Figure 4). Though heterogeneity between human and mouse cannot be ignored, mRNA vaccines encapsulated with nanocarriers may be prospective approaches to improve the clinical efficacy of ICIs in cervical cancer.

Bacterial such as *Listeria monocytogenes* (Lm) is another carrier type for the presentation of HPV antigens. Lm‐based therapeutic vaccines can activate both CD8^+^ T cells through MHC class I way and CD4^+^ T cells via secretion of cytokines (Chávez‐Arroyo & Portnoy, 2020). The safety of Lm‐E7 vaccine had been confirmed in a phase I clinical trial (Maciaga et al., 2009). Furthermore, ADXS11‐001, the E7 protein vaccines based on Lm vectors were well‐tolerated in participants and showed 1‐year overall survival (OS) of 36% in a phase II trial (Petit & Basu, 2013). Similar study was reported by Chowdhury et al, they found that *Escherichia coli* vector together with nano‐based CD47 antagonist successfully promoted tumor regression and extended mice survival by stimulating the infiltration of T lymphocytes in tumor sites (Chowdhury et al., 2019). Based on the results above, nanomaterials engineered Lm may also possible to be investigated to promote antigens presentation and specific cytotoxicity of therapeutic vaccines in cervical cancer.

## 5 CONCLUSIONS AND FUTURE PERSPECTIVES

There is an urgent need to develop novel therapeutic strategies that are tumor types specific and by‐effects limited for cervical cancer. Though nanocarriers based drug administration like liposomes, polymers, dendrimers, and inorganic materials have displayed great prospect of clinical transformation, it is almost impossible for single kind of nanoparticles to address all the difficulties such as dose‐dependent toxicities, drug biocompatibility, and controllable release during drug delivery in vivo. Therefore, developing the most appropriate multifunctional materials might be a promising choice for the effective delivery of anti‐cancer drugs that inhibit cell proliferation or induce cell killing through specifically targeting apoptosis pathway of cervical cancer. Considering that radiation is of crucial importance in cervical cancer treatment, it is clinically meaningful to develop nanoparticle‐based radiosensitizer combined with HPV antigens or newly identified tumor neoantigens in the near future. The effectiveness of lipidic nanocarriers in CRISPR‐E6/E7 editing system has been successfully verified in mouse model, the challenges ahead are thus to determine whether it is as powerful and safety in cervical cancer patients as that of in lung cancers. Although effective in treating precancers, therapeutic DNA vaccines targeting HPV E6/E7 cannot bring about clinical benefits for cervical cancer that has already occurred. In view of this, more powerful mRNA vaccines deserve to be exploited as the shortcomings such as single‐stranded instability and delivery inefficiency have been improved by advanced manufacturing technologies and appropriate nanocarriers.

## CONFLICT OF INTEREST

The authors have declared no conflict of interest for this article.

## AUTHOR CONTRIBUTIONS

**Peijie Zhou:** Funding acquisition; writing‐original draft; writing‐review & editing. **Wei Liu:** Data curation; funding acquisition. **Yong Cheng:** Supervision; validation. **Dong Qian:** Project administration; supervision; validation.

## RELATED WIREs ARTICLES

Virus‐like particles for vaccination against cancer

## Data Availability

There is no data are available in this review article.
